# Are multidose drug dispensing systems initiated for the appropriate patients?

**DOI:** 10.1007/s00228-018-2478-5

**Published:** 2018-05-16

**Authors:** Bram J. Mertens, H. F. Kwint, Rob J. van Marum, Marcel L. Bouvy

**Affiliations:** 1 Division of Pharmacoepidemiology and Clinical Pharmacology, Utrecht Institute for Pharmaceutical Sciences, Utrecht University, Universiteitsweg 99, 3584 CG Utrecht, The Netherlands; 2 SIR Institute for Pharmacy Practice and Policy, Leiden, Theda Mansholtstraat 5b, 2331 JE Leiden, The Netherlands; 3 Geriatric Department, Jeroen Bosch Hospital, ‘s-Hertogenbosch, The Netherlands; 4 Department of General Practice and Elderly Care Medicine and EMGO Institute for Health and Care Research, VU University Medical Center, Van der Boechorststraat 7, 1081 BT Amsterdam, The Netherlands

**Keywords:** Adherence, Dosing aids, Multidose drug dispensing, Community pharmacy, Health services

## Abstract

**Purpose:**

It is unknown if multidose drug dispensing (MDD) systems are initiated for the appropriate patients. Therefore, the objective of this study was to compare the medication management problems of patients who were about to start with a MDD system (MDD patients) and patients who continued manually dispensed medication (non-MDD users) in order to identify if the appropriate patients receive a MDD system.

**Methods:**

Patient interviews (semi-structured) were conducted by 44 community pharmacists at the patient’s home. Patients over 65 years of age, home dwelling and using at least five chronic drugs, were eligible for the study. An assessment tool was developed including 22 potential medication management problems, covering four domains: functional (7), organizational (7), medication adherence (6), and medication knowledge (2). Median scores were calculated with the interquartile range. Additionally, cognitive function was assessed with the Mini-Cog and frailty using the Groningen Frailty Indicator.

**Results:**

One hundred eighty-eight MDD users and 230 non-MDD users were interviewed. MDD users were older, more often female, and using more drugs. Forty-two percent of the MDD users were possibly cognitively impaired and 63% were assessed as frail compared to 20 and 27% respectively of the non-MDD users. MDD users had more potential organizational problems (3 vs. 1; *p* < 0.01), functional problems (2 vs. 1; *p* < 0.01), medication adherence problems (1 vs. 0; *p* < 0.01), and medication knowledge problems (1 vs. 0; *p* < 0.01) compared to non-MDD users. Seventy percent of the MDD users scored six or more potential medication management problems while this was 22% among non-MDD users.

**Conclusions:**

The majority of MDD systems were initiated for patients who experienced multiple potential medication management problems suggesting a decreased medication management capacity.

**Electronic supplementary material:**

The online version of this article (10.1007/s00228-018-2478-5) contains supplementary material, which is available to authorized users.

## Introduction

It is estimated that medication adherence to long-term therapies lies around 50% [1]. Non-adherence can be caused by a wide variety of reasons which can be divided into two major subgroups: intentional and unintentional non-adherence. Intentional non-adherent patients consciously decide to adapt their medication regimen. Unintentional non-adherent patients unconsciously fail to adhere to their medication regimen [2]. The capacity to adhere to the medication regimen may be diminished by a wide variety of reasons, e.g., a decline in cognitive function, complex dosing regimens, a change in appearance of outer packaging, and impaired manual dexterity [3, 4].

Intentional non-adherence is best targeted with multiple component interventions focused on patient education and patient’s behavior [2]. For unintentional non-adherence, more practical interventions may be more appropriate [2, 3, 5]. Especially older adults with polypharmacy and unintentional non-adherence might benefit from dosing aids. Dosing aids are intended to support patients with their medication use and improve the patient’s medication adherence [3, 6–8]. However, there are also concerns about the quality of drug treatment in patients who use a multidose drug dispensing (MDD) system. MDD users had a higher number of potential inappropriate medications and a reduced number of drug regimen changes [9–11].

If patients have lost the capacity to manage their medication, health care professionals can decide to dispense the medication via a dosing aid. As the number of older adults with polypharmacy is increasing, the use of dosing aids is also rising. In the Netherlands, automated MDD is preferably used when dosing aids are initiated by health care providers [12]. In a MDD system, all oral chronic medication intended for one dosing moment is electronically packed in plastic disposable bags. The content, patient information, and designated time of intake are printed on the bag [13].

MDD systems are relatively expensive compared to manual dispensing and should therefore be reserved for patients who have a decreased medication management capacity [12]. However, an unambiguous definition of a decreased medication management capacity is lacking. In practice, a proposal to initiate a MDD system can be suggested by a variety of people (e.g., the patients, relatives, nurses, physicians, or pharmacists). MDD systems are only reimbursed if the GP agrees to start a MDD system. Various assessment tools have been evaluated in a systematic review, but none were recommended to be used in daily practice because of a low reliability and validity [14]. As a consequence, no guidelines or assessment tools are available for health care professionals to identify patients with a decreased medication management capacity. Whether MDD systems are initiated in the appropriate patients is therefore unknown. The objective of this study was to compare potential medication management problems of patients who were about to start with a MDD system and patients who used manually dispensed medication in order to identify if the appropriate patients receive a MDD system.

## Method

### Study design

This was a case-control study performed in 44 community pharmacies in the Netherlands. The study was performed as part of a post academic training program in which the pharmacists participated. Eligible patients had to be over 65 years of age, home dwelling, and using a minimum of five chronic prescription drugs. Patients receiving their medication already via a MDD system and patients receiving professional home care responsible for the medication administration were excluded. MDD users (cases) were scheduled to start a MDD system, but at the time of the interview still used manually dispensed drugs. Non-MDD users (controls) used manually dispensed drugs. Controls were selected by the participating pharmacies. Community pharmacists selected patients who met the inclusion criteria from their pharmacy information system. Thereafter, a random selection of ten patients was made using computer-generated random numbers. From these ten patients, pharmacists invited patients to participate until at least five control patients were included. If pharmacists were not able to include five eligible MDD patients, additional non-MDD users could be included. A teleconference was organized to inform participating pharmacists and ensure consistency during the interviews. Pharmacists received detailed instructions on how to conduct the semi-structured interview and avoid closed-ended questions during the interview [20]. Additionally, pharmacists were instructed to inform the GP, after the patient’s approval, in case any relevant health complaint was suspected during the interview. Patients were invited to participate by telephone by the pharmacist. Interviews were conducted at the patient’s home.

### Medication management capacity

Existing tools which assessed the patient’s medication management capacity were used to develop a comprehensive assessment tool [15–19]. Existing tools use various strategies to assess the patient’s medication management capacity including self-reported adherence, the ability to open medication vials, the ability to read and interpret medication labels, and the ability to identify the medication in use. The design of the questionnaire was an iterative process of discussing and adjusting the questions. The first version of the questionnaire contained all used question from existing tools that assessed the patient’s medication management capacity. The questionnaire was supplemented with questions based on the Dutch guideline for patients using dosing aids [12]. The questionnaire was then tested in a pilot among current MDD users and controls. Undistinctive questions were deleted. Ambiguous questions were altered and clarified. Remaining questions were again tested in a pilot among current MDD users and non-MDD users. The final assessment tool contained 22 questions which covered four different domains: functional problems (7 questions), organizational problems (7 questions), adherence (6 questions), and medication knowledge (2 questions) (see Appendix 1). Answers were also considered correct if patients could not recall the exact indication (e.g., heart failure or myocardial infraction) but were able to give a more global indication (e.g., “for the heart”) [13]. Questions were dichotomized and each scored 1 point if present. All interviews were conducted by the community pharmacist. During the interview, patients were asked about their opinion on different aspects of their capacity to manage their medication. If present, the patient’s relative was also asked one question about their opinion on the patient’s capacity to manage their medication. Additionally, the patient’s GP was always asked about their opinion on the patient’s capacity to manage his medication.

### Cognitive function

The Mini-Cog was applied to explore the patient’s cognitive function. The Mini-Cog consists of a short memory test and a clock-drawing test (Appendix 2) [21]. A low score (≤ 2 points) indicates possible cognitive impairment. The Mini-Cog is easy to conduct and scored high on sensitivity and specificity compared to the MSSE [22].

### Frailty

The GFI was applied to assess patient’s frailty (Appendix 3) [23, 24]. The GFI consists of 15 questions, which can all be scored 1 point. If scored 4 or more points, patients were considered frail.

### Ethics and confidentiality

This study is in conformance with the Dutch Medical Research Involving Human Subjects Act (WMO). No formal ethical approval was needed to perform the study. The research protocol was approved by the Institutional Review Board of UPPER, Division of Pharmacoepidemiology and Clinical Pharmacology, Utrecht University [25]. Patients signed informed consent before the interview was performed. In order to protect the patient’s privacy, only age, gender, and the pharmacy’s study number were documented. Patients were assigned a unique study number (based on the pharmacy study number, age, and consecutive number of inclusion). Pharmacy study numbers were unknown to the researchers, only to a study coordinator (third party) who did not have access to the questionnaire data. In contrast to the researchers, the study coordinator was able to link the study number to the unique internal pharmacy number in order to check for double patients. The researchers could only contact pharmacies through the study coordinator.

### Sample size calculation and statistical analysis

A mean number potential problems of 10.5 (SD 5.01) was found in the pilot study. To demonstrate an absolute difference between the two groups of 20% in the number of potential problems with a power of 80% and an alpha of 0.05, 90 patients in both groups were needed. In order to maintain the possibility to stratify on different patient characteristics (e.g., cognition and frailty) and to compensate for the possible dropout of pharmacists and potential issues with unevaluable data, we aimed to include 440 patients. All data were analyzed using statistical software (SPSS version 23.0; SPSS Inc., Chicago, IL, USA). Descriptive statistics were used for basic characteristics. Median scores with the interquartile range are presented. Continuous variables were tested with a Mann-Whitney *U* test. Dichotomous variables were tested using a Pearson chi-square test. A Fleiss’ kappa coefficient was calculated for inter-rater agreement between the estimation of the patient’s relative and GP. The cutoff value for a decreased medication management capacity was based on the optimal ROC curve.

## Results

A total number 418 patients were included in the final analysis (188 MDD users and 230 non-MDD users). Fifteen included patients were excluded from the analysis because inclusion criteria were not met. MDD users were more often female, older, and using more drugs compared to non-MDD users (see Table 1).

**Table 1:** Basic characteristics

	MDD users (*n* = 188)		Non-MDD users (*n* = 230)		*p* value
Female, *n* (%)	126	67	121	53	0.003^a^
Age, median (IQR)	80.5	76–85	76	71–81	< 0.001^b^
Number of drugs per patient, median (IQR)	8	7–11	7	6–9	< 0.001^b^
Number of manually dispensed drugs per patient, median (IQR)	1	0–3	7	6–9	< 0.001^b^
Number of drugs in MDD, median (IQR)	7	5–8	–	–	–
Cognitive function and frailty					
Mini-Cog score ≤ 2, *n* (%)	78	42	45	20	< 0.001^a^
GFI score ≥ 4, *n* (%)	118	63	62	27	< 0.001^a^
Potential medication management problems					
Functional problems, median (IQR)	2	1–4	1	0–2	< 0.001^b^
Organizational problems, median (IQR)	3	2–4	1	1–2	< 0.001^b^
Adherence, median (IQR)	1	0–2	0	0–1	< 0.001^b^
Medication knowledge, median (IQR)	1	0–2	0	0–0	< 0.001^b^
Total potential medication management problems, median (IQR)	8	5–10	3	2–5	< 0.001^b^

*IQR* interquartile range, *MDD* multidose drug dispensing, *GFI* Groningen Frailty Indicator

^a^Tested with Pearson chi-square test

^b^Tested with Mann-Whitney *U* test

MDD users had more potential medication management problems (median 10; IQR 6–13) compared to the non-MDD users (median 3; IQR 2–5). Potential medication management problems were more prevalent among MDD users on all four domains as shown in Table 1. The cumulated score on all questions per patient is graphically presented in Appendix 4. Scores on separate questions are shown in Appendix 1.

Among MDD users, 70% (*n* = 132) had six or more potential problems. From MDD users with less than six potential problems (*n* = 56), only 34% (*n* = 19) of the patients or their relative and 52% (*n* = 29) of the GPs were of the opinion that the patient had lost the capacity to manage his medication (Table 2).

**Table 2:** The number of patients with a decreased capacity to manage their medication according to the patient or their relative and the GP

Non-MDD users	< 6 problems *n* = 180 (78%)	≥ 6 problems *n* = 50 (22%)	*p* value
Estimation by the patient or their relative, *n* (%)	4 (2%)	15 (30%)	< 0.001
Estimation by the GP, *n* (%)	2 (1%)	9 (18%)	< 0.001
MDD users	< 6 problems *n* = 56 (30%)	≥ 6 problems *n* = 132 (70%)	*p* value
Estimation by the patient or their relative, *n* (%)	19 (34%)	92 (70%)	< 0.001
Estimation by the GP, *n* (%)	29 (52%)	112 (85%)	< 0.001

*MDD* multidose drug dispensing, *GP* general practitioner

Among non-MDD users, 22% (*n* = 50) of the patients scored six or more potential problems. Within this group, 30% (*n* = 15) of the patients or their relative and 18% (*n* = 9) of the GPs were of the opinion that the patient had lost the capacity to manage their medication (Table 2).

Forty-two percent of the MDD users were assessed as cognitively impaired against 20% of the non-MDD users (OR 2.9; 1.9–4.5). Cognitively impaired patients had more potential medication management problems compared to patients with no cognitive impairment (median scores 8 vs. 4; *p* < 0.01). MDD users were assessed more often frail compared to non-MDD users (OR 4.3; 2.7–6.3). Frail patients had more potential medication management problems compared to non-frail patients (median scores 8 vs. 4; *p* < 0.01).

Using a different cutoff value of 5 or 7 did not substantially change the number of patients who were classified as receiving their medication via an inappropriate method of dispensing. A cutoff value of 5 resulted in a sensitivity of 74% and specificity of 67%. A cutoff value of 6 resulted in a sensitivity of 70% and specificity of 78%. A cutoff value of 7 resulted in a 60% sensitivity and specificity of 84%. Inter-rater agreement between relatives and GPs was high (*k* = 0.898).

## Discussion

This study showed that the majority of MDD systems are initiated for patients who are likely to have a decreased medication management capacity. Potential medication management problems were more prevalent among MDD users compared to non-MDD users and were related to all different domains (functional, organizational, adherence, and medication knowledge). MDD users were also older, used more medication, and were more often cognitively impaired and frail.

On the other hand, there is also a non-negligible group of MDD users (30%) for whom the start of a MDD system might be inappropriate. These patients had five or even less potential medication management problems. Among these patients, the majority estimated they had not lost the capacity to manage their medication. Although the initiation of a MDD system in these patients seems questionable, some patients may still need a MDD system because of a specific problem. For instance, patients who lost the ability to identify their medication correctly may not be capable to adhere to a complex medication regimen. With the use of a MDD system, this specific problem might be overcome.

Opposite of the possible overuse of MDD systems, also, underuse probably occurs. Twenty-two percent of the non-MDD users had six or more potential medication management problems. From these patients, about one-third indicated indeed to have lost the capacity to manage their medication. To identify these patients, health care professionals must screen the medication management capacity among older adults with polypharmacy, especially if they are cognitively impaired or frail.

The development of an assessment tool to identify patients with a decreased medication management capacity is difficult as a gold standard is lacking. In our study, we used a score of 6 or more potential medication management problems as an indicator that the patient had a decreased medication management capacity. This cutoff value remains arbitrary as there is no objective method to assess the medication management capacity. Using different cutoff values resulted in a 10% increase or decrease of patients who received the appropriate method of dispensing. Using a higher cutoff value of 7 would result in more MDD users for whom a MDD system might be inappropriate. But on the other hand, less non-MDD users should receive a MDD system.

More importantly, when choosing between MDD and manual dispensing, health care professionals have to balance the patient’s self-efficacy, patronizing patients, the higher costs, and the patient’s medication management capacity. As shared decision-making is expected to improve medication adherence, the patient’s opinion must be taken into account in the decision to start a MDD system [26, 27]. MDD systems must not be initiated in patients who do not agree with the initiation of a MDD system, even if multiple medication management problems are prevalent. On the other hand, health care professional must be able to initiate a MDD system in patients who state they have lost the capacity to manage their medication but only experience a few medication management problems. The proposed questionnaire is therefore merely intended to support health care professionals to identify patients with a potentially decreased medication management capacity. It must explicitly not be decisive in the decision to start a MDD system.

This study was the first in which patients were included who were about to start with a dosing aid, but at the time of the interview still used manually dispensed medication. This enabled us to compare medication management problems between a high-risk population (MDD users) and a randomly selected population (non-MDD users). Because 44 different community pharmacies throughout the Netherlands participated in the study, the results are well generalizable to the Dutch population. A third strength of the study was the comprehensive character of the questionnaire. The questionnaire was developed with a broad scope combining different elements which might be of influence on the patient’s medication management capacity. Additionally to the questionnaire, the patient or their relative and the GP were asked about their opinion of the patient’s capacity to manage his medication. As a result, three different signs for a decreased medication management could be combined, firstly the start of a MDD system, secondly the score on the questionnaire, and thirdly the patient’s or their relative’s estimation.

The study also had some limitations. Firstly, MDD users were invited to participate in the study if the decision was already made to start a MDD system. As a consequence, patients or their relatives and GPs might be inclined to confirm that the patient lost the capacity to manage his medication. This bias was only present in MDD users and not among non-MDD users as they were randomly selected. Among the non-MDD users, the opposite might be the case, GPs could be unaware of a possible decreased medication management capacity. Secondly, basic characteristics between the MDD users and non-MDD users did not match on age, gender, and number of drugs. Non-MDD users were deliberately not matched on these characteristics, as these characteristics might be a determinant for a decreased medication management capacity. Thirdly, patients with homecare responsible for the medication administration were excluded as Dutch guidelines state that patients receiving homecare should always receive medication via a MDD system. Especially, these patients are expected to have multiple medication management problems. Including this group would probably result in even more MDD users with multiple medication management problems. Finally, in this study, potential medication management problems leading to the start of a MDD system were identified, whether the start of a MDD system resolved these problems is unknown. This must be elucidated in further research.

## Conclusions

For the majority of MDD users, the initiation of a MDD system was appropriate as these patients experienced multiple potential medication management problems suggesting a decreased medication management capacity. However, for a minority of MDD users, the start of a MDD system might be questionable according to the limited number of medication management problems. On the other hand, there is a group of non-MDD users who seem to have a decreased medication management capacity, but still use manually dispensed medication. These patients might benefit from a MDD system. To identify these patients, health care professionals must screen older patients with polypharmacy on their medication management capacity.

## Electronic supplementary material

ESM 1 (DOCX 20.5 kb)

ESM 2 (DOCX 29.5 kb)

ESM 3 (DOCX 17.1 kb)

ESM 4 (DOCX 19.9 kb)
