# Supplementary material for: Are multidose drug dispensing systems initiated for the appropriate patients?
Source: Eur J Clin Pharmacol. 2018 May 16;74(9):1159–64. doi: 10.1007/s00228-018-2478-5 (PMC6096704; doi:10.1007/s00228-018-2478-5)
Supplement: Supplementary file 1 — (DOCX 20.5 kb) [file 228_2018_2478_MOESM1_ESM.docx]

Appendix 1: Scores on all separate questions

|  |  | **MDD-users**  **(n=188)** | | **Non-MDD-users**  **(n=230)** | | |  |
| --- | --- | --- | --- | --- | --- | --- | --- |
| **Domain** | **Question** | **Yes** | **No** | **Yes** | **No** | **OR** | **CI 95%** |
| Adherence | Patient takes all medication on the designated time of intake |  | **62 (33%)** |  | **30 (13%)** | 3,3 * | 2,0 – 5,4 |
| Adherence | Patient takes medication of one dosing moment divided over the day | **46 (24%)** |  | **41 (18%)** |  | 1,5 | 0,9 – 5,4 |
| Adherence | Patient takes the right amount of medication each dosing moment |  | **19 (10%)** |  | **9 (4%)** | 2,7 * | 1,2 – 6,2 |
| Adherence | Patient takes the medication the right amount of times per day |  | **33 (18%)** |  | **21 (9%)** | 2,1 * | 1,2 – 3,7 |
| Adherence | Patient adheres to special instructions |  | **38 (21%)** |  | **22 (10%)** | 2,4 * | 1,4 – 4,3 |
| Adherence | Patient forgets medication on a regular basis | **83 (45%)** |  | **27 (12%)** |  | 5,9 * | 3,6 – 9,7 |
| Functional | Patient is able to read the label |  | **37 (20%)** |  | **14 (6%)** | 3,8 * | 2,0 – 7,2 |
| Functional | Patient uses supportive reading tools to read the label | **108 (57%)** |  | **141 (61%)** |  | 0,9 | 0,6 – 1,3 |
| Functional | Patient experiences problems in daily life because of poor vision | **47 (25%)** |  | **19 (8%)** |  | 3,7 * | 2,1 – 6,6 |
| Functional | Patient can correctly explain the information of a random chosen drug label |  | **37 (20%)** |  | **9 (4%)** | 6,0 | 2,8 – 12,8 |
| Functional | Patient has troubles with the packaging of the medication | **66 (35%)** |  | **39 (17%)** |  | 2,7 * | 1,7 – 4,2 |
| Functional | Patient finds it difficult to manage his drug regimen | **113 (26%)** |  | **26 (11%)** |  | 12,0 * | 7,3 – 19,9 |
| Functional | Patient has reduced manual dexterity | **81 (56%)** |  | **56 (24%)** |  | 2,4 * | 1,6 – 3,6 |
| Organisational | Patient finds it difficult to order the medication in time | **98 (52%)** |  | **29 (13%)** |  | 7,5 * | 4,7 – 12,2 |
| Organisational | Patient has medication at home for a period longer than three months | **45 (24%)** |  | **15 (7%)** |  | 4,5 * | 2,4 – 8,4 |
| Organisational | Patient stores his medication on multiple places | **80 (43%)** |  | **81 (35%)** |  | 1,4 | 0,9 – 2,0 |
| Organisational | Patient has multiple generic brands at home from one drug | **73 (39%)** |  | **66 (29%)** |  | 1,6 * | 1,1 -2,4 |
| Organisational | Patient stores his medication according to the storing conditions |  | **21 (11%)** |  | **7 (3%)** | 4,0 * | 1,6 – 9,5 |
| Organisational | Patients has ceased medication at home | **95 (51%)** |  | **56 (24%)** |  | 3,2 * | 2,1 – 4,8 |
| Organisational | Patient finds it difficult when the appearance of a drug changes | **116 (62%)** |  | **110 (48%)** |  | 1,8 * | 1,2 – 2,6 |
| Medication knowledge | Patient can recall the correct indication of > 75% of their medication |  | **97 (52%)** |  | **35 (15%)** | 5,9 * | 3,8 – 9,4 |
| Medication knowledge | Patients designates all medication correctly |  | **108 (58%)** |  | **35 (16%)** | **7,5 *** | 4,7 – 11,9 |
| ^*^ represent significant values with a p-value of < 0.05 | |  |  |  |  |  |  |
