# Supplementary material for: Are multidose drug dispensing systems initiated for the appropriate patients?
Source: Eur J Clin Pharmacol. 2018 May 16;74(9):1159–64. doi: 10.1007/s00228-018-2478-5 (PMC6096704; doi:10.1007/s00228-018-2478-5)
Supplement: Supplementary file 2 — (DOCX 29.5 kb) [file 228_2018_2478_MOESM2_ESM.docx]

Appendix 2: Mini-Cog

**possibly cognitively impaired**

Mini-Cog

3-item recall = 0

3-item recall = 1,2

3-item recall = 3

CDT abnormal

CDT normal

**possibly cognitively impaired**

**Probably no cognitive impairment**

**Probably no cognitive impairment**

Figure 1: algorithm of the Mini-Cog. Items to be recalled: apple, coin and table. CDT: clock drawing test. Adapted from Borson et al.
