# Supplementary material for: Are multidose drug dispensing systems initiated for the appropriate patients?
Source: Eur J Clin Pharmacol. 2018 May 16;74(9):1159–64. doi: 10.1007/s00228-018-2478-5 (PMC6096704; doi:10.1007/s00228-018-2478-5)
Supplement: Supplementary file 3 — (DOCX 17.1 kb) [file 228_2018_2478_MOESM3_ESM.docx]

Appendix 3: Groningen Frailty Indicator

| Number | **For questions 1 -4: Is the patient able to carry out these tasks single handed without any help?** (The use of help resources such as walking stick, walking frame, wheelchair, is considered independent) |
| --- | --- |
| 1 | Shopping |
| 2 | Walking around outside (around the house or to the neighbours) |
| 3 | Dressing and undressing |
| 4 | Going to the toilet |
|  |  |
| 5 | What mark does the patient give himself/herself for physical fitness? (scale 0 to 10) |
| 6 | Does the patient experience problems in daily life due to poor vision? |
| 7 | Does the patient experience problems in daily life due to being hard of hearing? |
| 8 | During the last 6 months has the patient lost a lot of weight unwillingly?  (3 kg in 1 month or 6 kg in 2 months) |
| 9 | Does the patient take 4 or more different types of medicine? |
| 10 | Does the patient have any complaints about his/her memory? |
| 11 | Does the patient sometimes experience an emptiness around him/her? |
| 12 | Does the patient sometimes miss people around him/her? |
| 13 | Does the patient sometimes feel abandoned? |
| 14 | Has the patient recently felt downhearted or sad? |
| 15 | Has the patient recently felt nervous or anxious? |
|  |  |
|  | **Sum score:** |
|  |  |
|  | **Scoring** |
|  | *Questions 1–4:* Independent = 0; dependent = 1 |
|  | *Question 5:* 0–6 = 1; 7–10 = 0 |
|  | *Questions 6–9:* No = 0; yes = 1 |
|  | *Question 10:* No and sometimes = 0; yes = 1 |
|  | *Questions 11–15:* No = 0; sometimes and yes = 1 |

Figure 2: Groningen Frailty Indicator. Adapted from Schuurmans et. al.
