# Supplementary material for: Are multidose drug dispensing systems initiated for the appropriate patients?
Source: Eur J Clin Pharmacol. 2018 May 16;74(9):1159–64. doi: 10.1007/s00228-018-2478-5 (PMC6096704; doi:10.1007/s00228-018-2478-5)
Supplement: Supplementary file 4 — (DOCX 19.9 kb) [file 228_2018_2478_MOESM4_ESM.docx]

Appendix 4:

Number of patients with scores on all potential medication management problems
